# Identification of Key Genes Related to Skin Burns Based on Bioinformatics Analysis

**DOI:** 10.1093/jbcr/irac132

**Published:** 2022-09-15

**Authors:** Boheng Zhu, Gaofei Zhang, Wuquan Li, Wende Cao, Jinglin Zhang, Hong Wang

**Affiliations:** Department of Burns, The Second Affiliated Hospital of Kunming Medical University, Kunming, China; Department of Burns, The Second Affiliated Hospital of Kunming Medical University, Kunming, China; Department of Burns, The Second Affiliated Hospital of Kunming Medical University, Kunming, China; Department of Burns, The Second Affiliated Hospital of Kunming Medical University, Kunming, China; Department of Burns, The Second Affiliated Hospital of Kunming Medical University, Kunming, China; Department of Burns, The Second Affiliated Hospital of Kunming Medical University, Kunming, China

## Abstract

To further understand the regulatory network and molecular mechanisms of gene expression after skin burns, we performed bioinformatics analysis of gene expression profiles of skin burn samples and identified key genes associated with skin burns. The GSE8056 and GSE139028 datasets were downloaded from the Gene Expression Omnibus database for analysis and validation. The limma package was used to screen for differentially expressed genes (DEGs). Gene ontology and pathway enrichment analyses (KEGG) were then performed. Subsequently, LASSO regression analysis was performed on DEGs and a regulatory network map of skin burn-related genes was constructed. Finally, the infiltration of immune cells was calculated and coexpression network maps of immune-related key genes and skin regeneration genes were constructed. Analysis of the GSE8056 dataset showed that 432 genes were upregulated and 351 genes were downregulated. The DEGs were mainly focused on immune response and skin regeneration. Meanwhile, these two groups of pivotal genes were significantly associated with abnormal infiltration of nine immune cells. GSE139028 validation revealed that three hub genes associated with skin burn immunity were differentially expressed, except for S100A8, while only the DPT gene was differentially expressed among the seven hub genes associated with skin regeneration. In short, the effect of skin burn on patients is to regulate the expression of immune-related genes UPP1, MMP1, MMP3, and skin regeneration-related gene DPT, which may be the key target for the treatment of skin burn.

Skin burns are a common skin trauma with a high incidence. With the development of modern civilization, multiple factors can cause human skin burns.^1^ Skin burn trauma is not only a simple pathophysiological process, but also a destructive injury that causes many structural and functional defects in organ systems. It is one of the most devastating diseases in the world.^2^ Skin burns will change metabolic balance, immune response, and tissue structure, and these changes will cause a chain reaction in patients, including wound infections, respiratory failure, and other diseases.^3,4^ According to the degree of skin burn, the physiological and morphological changes are also different, and the hypermetabolic response can effectively reflect the degree of burn.^5,6^ In addition, skin regeneration after skin burn is related to patients’ physical and mental health problems. Skin regeneration is closely related to the changes in internal environment after burn, and the study of postburn tissue may help patients recover after burn. In vitro stem cell research on postburn skin tissue found that undamaged skin stem cells can be extracted from burned skin tissue, which will provide an ideal cell source for skin regeneration.^7^ In vivo animal model experiments show that different colors of light have effects on angiogenesis of skin tissue and myofibroblasts at wound after skin burn.^8^ However, few studies have studied the molecular mechanism of skin regeneration in postburn tissues.

Immune mechanism plays an important role in the treatment of skin burns. Burns cause damage to the body’s defense system, including loss of the skin barrier and increased necrotic tissue, which makes patients susceptible to infection. Malignant wound infection and sepsis are the main causes of death.^9,10^ Studies have shown that the body produces immune suppression after burns, and this immune suppression is related to the degree of skin damage.^11^ After severe skin burns, the chemotaxis of phagocytes is impaired, the chemotaxis of neutrophils is weakened, and the phagocytosis and intracellular killing effect are weakened, which leads to specific immune system suppression. It is currently believed that the synthesis of inflammatory mediators after burns can change the function of macrophages, but the mechanism is not clear.^12^ In addition, the immune system is focused on skin transplantation after burns. The immune system’s rejection of skin donors can cause transplantation failures and difficulties in donor selection.^9^

In recent years, with the development of second-generation sequencing and computer statistical analysis methods, it has become a trend to use bioinformatics methods to analyze new targets and related molecular pathways of the occurrence and development of diseases. For example, Gao *et al*. elucidate burn mechanisms through bioinformatics analysis of gene expression profiles and construction of immune-associated protein–protein interaction (PPI) network diagrams.^13^ Lu *et al*. screened differentially expressed genes (DEGs), key biological functions, and related pathways using a comprehensive bioinformatics approach that integrated gene expression profile data. In addition, a burn PPI network was constructed to identify potential burn biomarkers. These integrated findings further expand our understanding of the molecular mechanisms that regulate leukocyte responses to burns and may provide prospective targets for the development of novel therapeutic strategies.^14^ In this paper, dataset GSE805 was used for differential gene screening to obtain DEGs related to burns. Then gene ontology (GO), KEGG analysis, and LASSO regression analysis were performed to obtain key genes related to skin burns. Then SSGSEA was used to calculate immune infiltration of skin burns and normal tissues. Pearson correlation was used to analyze the correlation between key genes and 24 kinds of immune cells in skin burn patients. Finally, GSE139028 dataset was used for gene expression verification to obtain key skin regeneration-related genes related to skin burn.

## MATERIALS AND METHODS

### Data Sources and Target Information

Datasets GSE8056 and GSE139028 were downloaded from the Gene Expression Omnibus database (https://www.ncbi.nlm.nih.gov/geo/). GSE8056 contained transcriptome data of nine skin burn patients’ wound tissues (three cases 0–3 days, three cases 4–7 days, three cases >7 days) and three normal samples. All skin samples were obtained within a few minutes after they were removed from patients in the operating room, and burn samples were taken from the edge of the wound. After separation, RNA from five tissue specimens was pooled together at the same quality for each array replication. GSE139028 contained transcriptome data from six skin burn patients and three normal individuals, which was used for validation. This study does not involve human clinical data, which are publicly available and do not require ethical approval.

### Analysis of Differential Expression in Skin Burn Tissue

The LIMMA package is a tool for differential analysis of Nucleic Acids Research, published in 2015. The difference analysis compares the datasets of control samples (CK) and treatment samples (Treat) to obtain the DEGs between treatment samples and control samples. It helps to screen for mRNAs or signaling pathways that play a role in the burn process. In this study, the limma package was used to compare the gene expression differences between tissues of different skin burn time and normal sample tissues in the dataset GSE8056, including three sets of data of 0–3, 4–7, and >7 days after burn. If the filter condition is set to |log_2_FC|, the value must be greater than or equal to 1 and the *P* value must be less than 0.05. The analysis result uses the jvenn (http://jvenn.toulouse.inra.fr/app/example.html) online website to take the intersection of each group of differential genes.^15^

### GO and KEGG Pathway Enrichment Analysis

GO and KEGG analyses were performed using the clusterProfiler (version 3.10.1) package. The enrichplot and DOSE packages were used to supply enrichment result visualization to help interpretation. *P* < .05 and adjusted *P* < .05 were set as the threshold values.

### Lasso Regression Analysis

LASSO regression, also known as lasso regression, is a new variable selection technique proposed by Robert Tibshirani in 1996. LASSO is a shrinkage estimation method. Its basic idea is to minimize the residual sum of squares under the constraint that the sum of the absolute values of the regression coefficients is less than a constant, In this study, in order to screen out the pivotal genes related to immunity and skin regeneration after skin burns, the R glmnet package performed LASSO regression analysis on the DEGs related to skin burns.^16^

### Prediction of the Regulatory Network of Pivotal Genes

In the Network Analyst (https://www.networkanalyst.ca), analysis platform was used to predict the transcription factor (TF) regulatory network of pivotal genes. In addition, we apply miRWalk comprehensive gene database (http://mirwalk.umm.uni-heidelberg.de/) based on the hypothetical mechanism of competing endogenous RNAs (CeRNA) to predict miRNAs targeting pivotal genes related to skin burns, and then use Starbase database (http://starbase.sysu.edu.cn/) to predict the targeting relationship of miRNA-lncRNA, and use Pearson correlation analysis to screen out lncRNAs that are positively related to pivotal genes. All TF targeting relationships and lncRNA/miRNA/mRNA targeting relationships were constructed into a regulatory network diagram using Cytoscape.

### Correlation Between Pivotal Genes and Immune Infiltration

SSGSEA (single sample GSEA) was used to calculate immune cell infiltration in skin burn/normal samples.^17^ SSGSEA was used for immunoinvasion analysis of tumor samples. The immune cells or immune functions of each sample and the activity of immune pathways were obtained by SSGSEA, and then the samples were grouped according to the immune activity. SSGSEA calculated the rank value of each gene according to the expression profile file, and then carried out subsequent statistical analysis. The 24 immune-related gene sets include not only immune cell types, but also immune-related pathways and immune-related functions. These immune-related gene sets are very rich in content. Using these sets of immune-related genes for analysis, the immune activity of each sample can be accurately determined. R “ggplot2” and “ggpubr” packages are used to draw box diagrams and visualize the results. *t*-Test was used to compare the immune cell scores of each group with normal. Pearson correlation analysis was explored by the correlation between different immune cells and pivotal genes (including immune genes and skin regeneration pivotal genes), and heat map was used to describe the results.

### Gene Expression Verification

Pearson correlation was used to analyze pivotal immune-related genes and skin regeneration-related genes to construct a coexpression network. Then the dataset GSE139028 was used to verify the expression of pivotal genes, and the difference between the expression of each group of genes and the normal group was compared using the *t*-test.

## RESULTS

### Screening of Skin Burn-Related Genes

**Figure 1.**
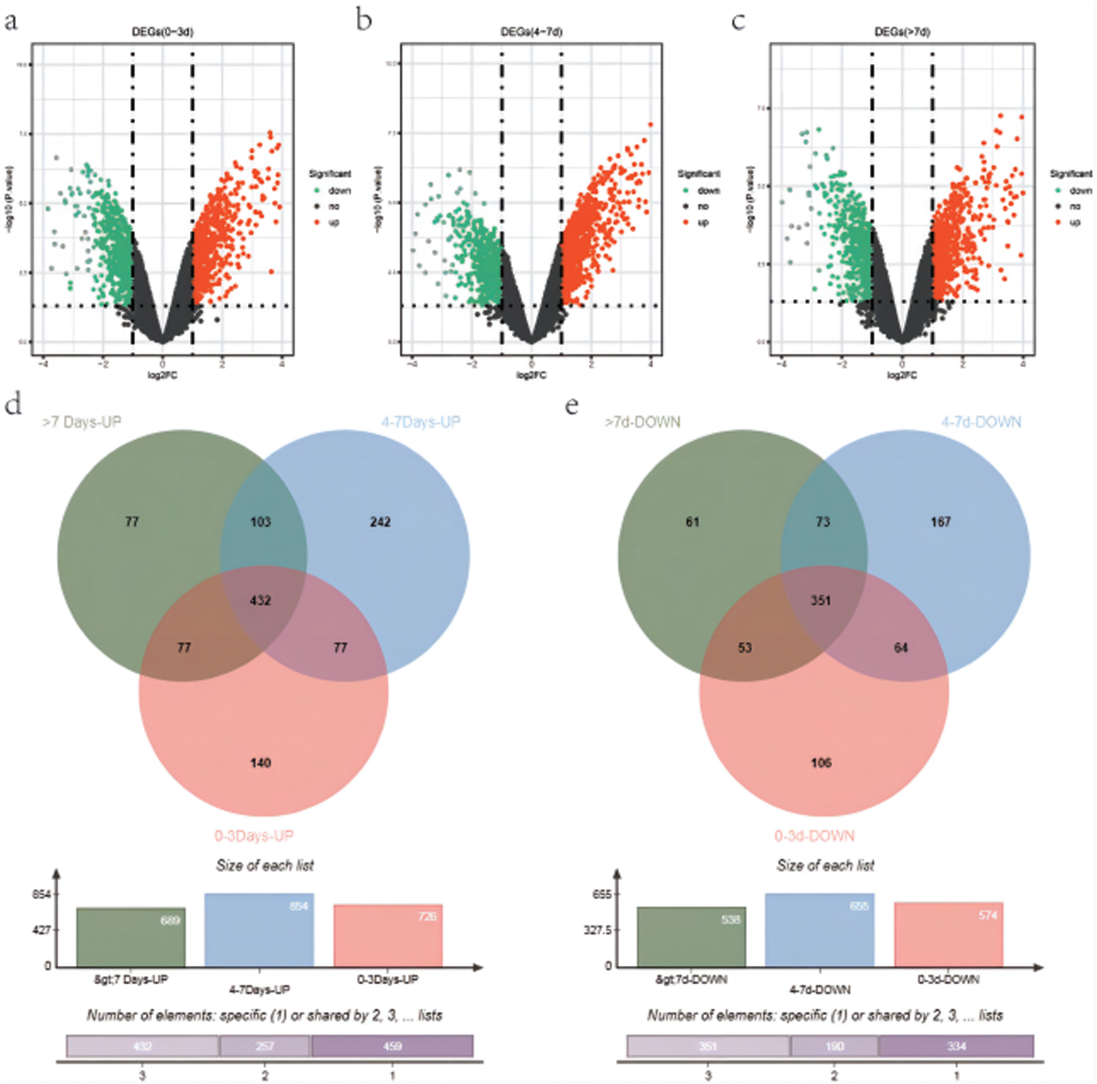
Screening of skin burn-related genes. (A–C) The volcanic map of differentially expressed genes between samples 0–3, 3–7, and >7 days after burn and normal samples. (D, E) The screening of upregulated and downregulated skin burn-related genes respectively. Green represents genes differentially expressed between >7 days after burn/normal tissues, blue represents genes differentially expressed between 4–7 days after burn/normal tissues, and pink represents genes differentially expressed between 0–3 days after burn/normal tissues.

The burn tissue samples in the dataset GSE8056 were divided into three groups: 0–3 , 4–7 and >7 days. The normal samples were used as the control. The results of differential gene analysis showed that there were 1300 DEGs in the sample group 0–3 days after burn, of which 726 genes were upregulated and 574 genes were downregulated (Figure 1A). There were 1509 DEGs in the sample group 4–7 days after burn, of which 854 genes were upregulated and 655 genes were downregulated (Figure 1B). There were 1227 DEGs in the sample group >7 days after burn, of which 689 genes were upregulated and 538 genes were downregulated (Figure 1C). Jvenn was used to select the intersection of upregulated and downregulated genes in three groups of samples. Finally, 432 genes were upregulated in all three groups of skin burn tissues, and 351 genes were downregulated in all three groups of skin burn tissues (Figure 1D and E).

### Functional Enrichment Analysis of Skin Burn-Related Genes

**Figure 2.**
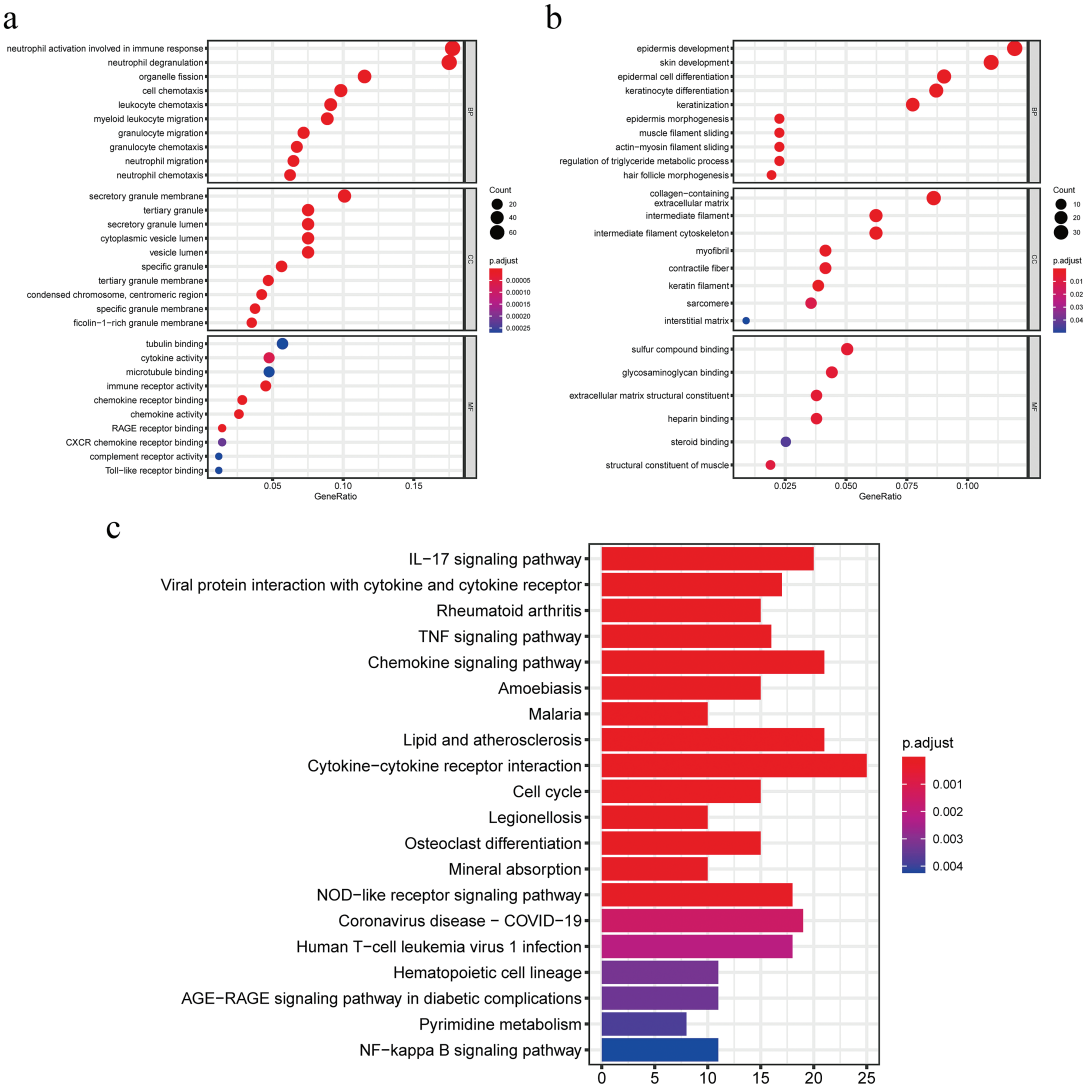
Go function analysis and KEGG pathway enrichment analysis of skin burn-related genes. (A, B) The Go function enrichment bubble diagram of upregulated and downregulated skin burn-related genes, the ordinate represents the enriched GO term, the circle size represents the skin burn-related genes enriched by the GO term, and the color from blue to red represents the reliability of the results from low to high. (C) The enrichment bar graph of upregulated skin burn-related gene KEGG pathway, the ordinate represents the enriched KEGG pathway, the length of the bar represents the number of differentially expressed genes enriched by the pathway, and the color from blue to red indicates that the reliability of the results is from low to high.

The biological processes are mainly enriched in immune response-related, including cell chemotaxis, organelle fission, neutrophil degranulation, neutrophil activation involved in immune response, and so on (Figure 2A). Among them, neutrophil activation involved in immune response process accounts for the largest proportion. In terms of molecular function, it is mainly related to the activity of cytokines, chemokines, and receptors, including Toll-like receptor binding, complement receptor activity, CXCR chemokine receptor binding, RAGE receptor binding, etc. The analysis results of downregulated genes after skin burn are shown in Figure 2B. The biological processes are mainly enriched in cell differentiation and skin and epidermal development, including keratinization, keratinocyte differentiation, epidermal cell differentiation, skin development, and epidermis development. Among them, the most enriched genes are in the process of epidermis development. In terms of molecular function, it is mainly related to muscle structure synthesis, heparin binding, and steroid binding, including structural constituent of muscle, steroid binding, heparin binding, extracellular matrix structural constituent, glycosaminoglycan binding, sulfur compound binding, etc. In summary, the GO analysis results show that the main upregulated genes after skin burns are immune-related genes, and the downregulated genes are mainly related to skin development and repair.

KEGG analysis showed that the main signal pathways involved in the upregulated differential genes after skin burn were significantly correlated with immune response, signal transduction, virus infection. and cell cycle-related pathways (Figure 2C). However, the downregulated skin burn-related genes in this study were not enriched into the KEGG pathway.

### Screening of Pivotal Genes Related to Skin Burn

**Figure 3.**
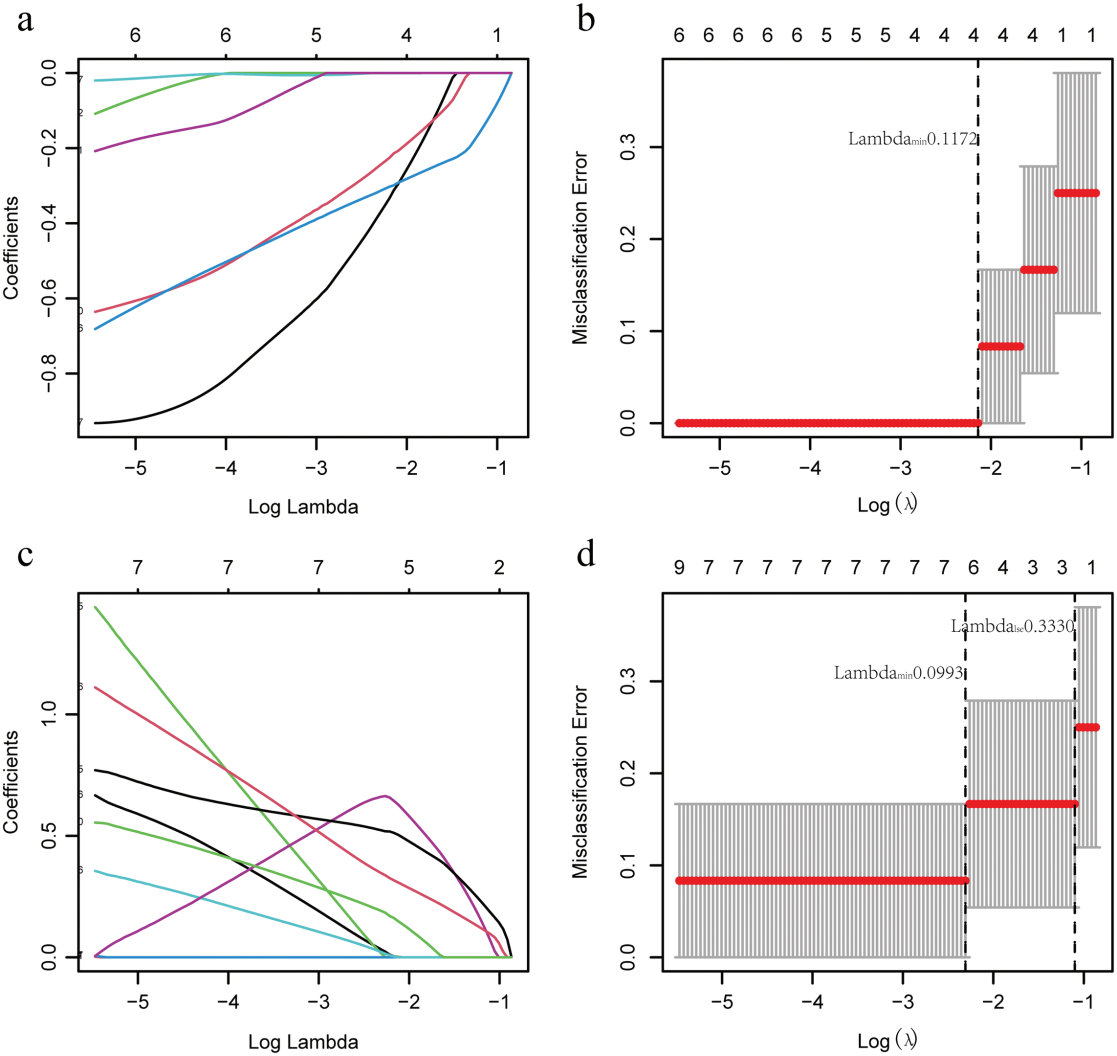
Screening of pivotal genes related to skin burn immune response and skin regeneration. (A, B) LASSO regression analysis to screen pivotal immune-related genes. (C, D) LASSO regression analysis to screen pivotal genes related to skin regeneration. The abscissa deviance represents the proportion of residual explained by the model, showing the variation relationship between the number of characteristic genes and the proportion of residual explained (dev), and the ordinate is the coefficient of genes (A and C). The abscissa is log (Lambda), and the ordinate represents the error of cross-validation (B and D).

Lasso regression analysis showed that when Lambda.min was 0.1172, four representative immune-related genes were found in 432 skin burn-related upregulated genes, S100A8, UPP1, MMP1, and MMP3, respectively (Figure 3A and B). Similarly, when Lambda.min was 0.0993, seven representative skin regeneration-related genes were found in 351 skin burn-related downregulated genes, namely NR3C2, HSPB3, DPT, TNNC1, TMEM255A, DKK2, and SERTM1(Figure 3C and D). Therefore, in the follow-up study, four immune-related genes and seven regeneration-related genes were defined as characteristic genes related to skin burn.

### Prediction of Regulatory Network of Pivotal Genes Related to Skin Burns

**Figure 4.**
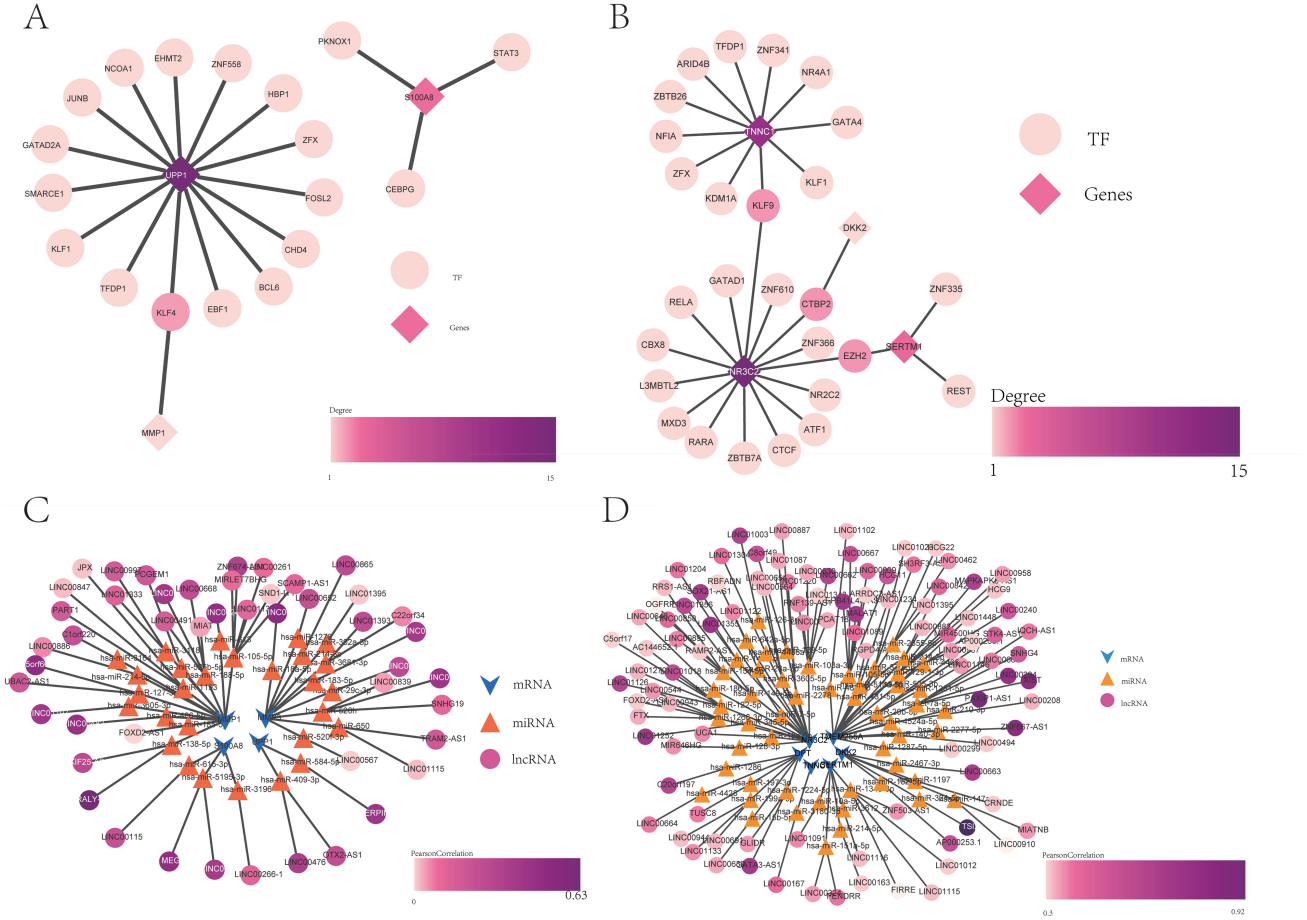
Prediction of TF and lncRNA-miRNA-mRNA regulatory networks of pivotal genes related to skin burn: (A) the immune-related pivotal gene TF regulatory network and (B) the TF regulatory network, a pivotal gene related to skin regeneration. Diamond represents pivotal genes related to skin burn, dot represents transcription factor (TF), and color from light to dark represents degree from small to large. (C) The lncRNA-miRNA-mRNA regulatory network of pivotal immune-related genes related to skin burns. (D) The lncRNA-miRNA-mRNA regulatory network of pivotal skin regeneration-related genes related to skin burn.

Network Analyst’s prediction of skin burn immune-related factors showed that UPP1 had 15 TFs, S100A8 had 3 TFs, and MMP1 had 1 TF (Figure 4A). KLF4 is a TF shared by UPP1 gene and MMP1. MMP3 did not predict TFs. Similarly, TF regulatory networks of skin regeneration-related genes were analyzed (Figure 4B). Among them, KLF9 is a common TF of TNNC1 gene and NR3C2 gene, CTBP2 is a common TF of DKK2 gene and NR3C2 gene, and EZH2 is a common TF of SERTM1 gene and NR3C2 gene. In addition, miRWalk and Starbase databases were used to construct lncRNA-miRNA-mRNA regulatory network (CeRNA network) of skin burn-related genes (Figure 4C and D). ALY-AS/has-miR-138-5p/MMP1 has the highest correlation in the CeRNA network of immune-related genes. In the CeRNA regulatory network of pivotal genes related to skin regeneration, except HSPB3, the other six pivotal genes are regulated by lncRNA and miRNA.

### Correlation Between Pivotal Genes and Immune Infiltration

**Figure 5.**
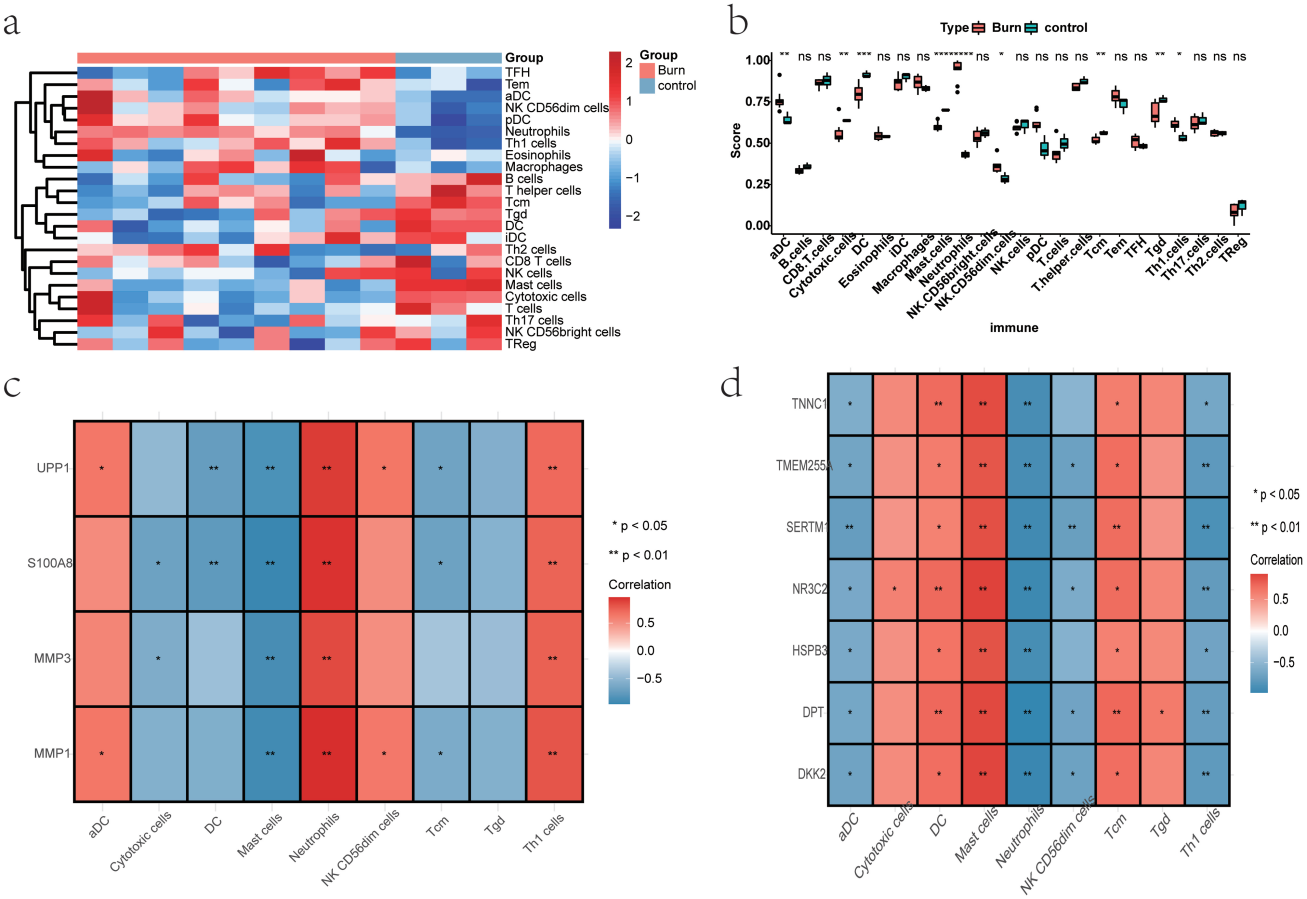
Correlation between pivotal genes and immune infiltration. (A) The heat map of different immune cell content in skin burns samples calculated by ssGSEA. (B) The box line diagram of differential immune cells between skin burn/ normal groups, blue represents the normal group and pink represents the skin burn group. (C, D) The correlation between skin burn-related immune response and pivotal genes of skin regeneration and immune cells, respectively. Red indicates positive correlation, blue indicates negative correlation, and the darker the color, the greater the correlation. All “*” indicates *P* < .05 and “**” indicates *P* < .01.

Twenty-four immune-related gene sets were used to calculate the infiltration of immune cells through ssGSEA (Figure 5A). At the same time, analysis of differences between groups found that immune cells Neutrophils, Mast cells, DC, Tcm, Cytotoxic cells, Tgd, aDC, Th1 cells, and NK CD56dim cells were significantly different between skin burn tissue and normal tissue (Figure 5B). Further explore the Pearson correlation between pivotal genes related to skin burns and nine different immune cell contents. Neutrophils, aDC, Th1 cells, and NK CD56dim cells have a strong positive correlation with pivotal immune-related genes related to skin burns (*P* < .05), and Mast cells, DC, Tcm, and Cytotoxic cells are related to pivotal immune-related genes related to skin burns genes have a strong negative correlation, while immune cell Tgd has no significant correlation with pivotal genes related to skin burns (Figure 5C). In addition, Mast cells, DC, Tcm, Tgd, and Cytotoxic cells have a strong positive correlation with pivotal skin regeneration-related genes related to skin burns (*P* < .05), while Neutrophils, aDC, Th1 cells, and NKCD56dim cells are related to skin burns. Related pivotal skin regeneration-related genes have a strong negative correlation (Figure 5D). In short, the results show that the pivotal genes related to immune response and skin regeneration after skin burns are closely related to immune cells.

### Pivotal Gene Expression Verification

**Figure 6.**
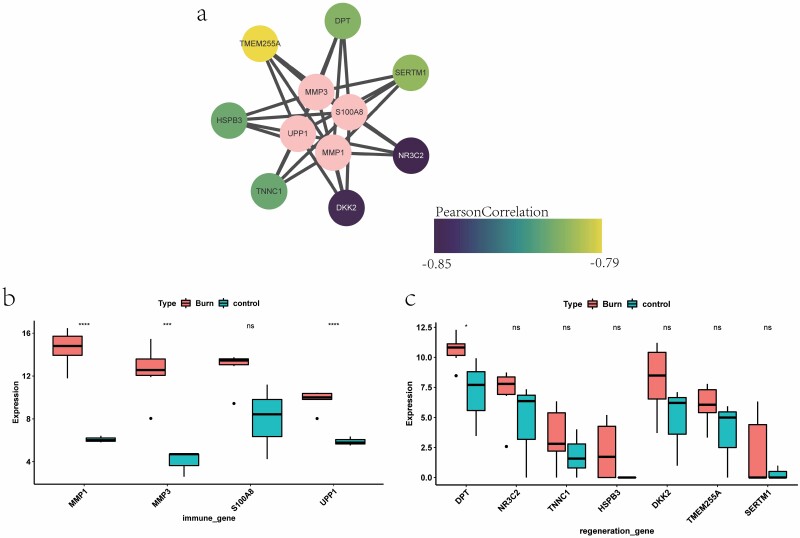
Expression verification of pivotal genes. (A) The coexpression network of skin burn-related pivotal skin regeneration genes and pivotal immune-related genes. The pink dots in the figure represent pivotal immune-related genes, and the other dots represent pivotal skin regeneration genes related to skin burn. (B, C) The expression of pivotal immune and skin regeneration-related genes related to skin burn, respectively. Pink represents skin burn tissue and blue represents normal samples.

First, a coexpression network of pivotal immune-related genes and skin regeneration genes was constructed by using Pearson correlation analysis (Figure 6A), and the results showed that there is widespread coexpression of pivotal genes. In addition, GSE139028 was used to reverify the expression of pivotal genes after skin burns. The results are shown in Figure 6B and C. Among the four pivotal immune-related genes, except for S100A8 in skin burn tissue and normal tissue, the expression difference is not significant, and the other three genes have significant differences. Among the seven pivotal genes related to skin regeneration, except for DPT, which is a gene that is significantly differentially expressed between skin burn tissue and normal tissue, the other genes are not significant.

## Discussion

Skin burn is the most common clinical disease. Its healing process generally includes three stages: inflammatory reaction, tissue regeneration, and remodeling. However, the relevant mechanism is not completely clear. First, the dataset GSE8056 was used to analyze the data of 0–3, 4–7, and >7 days after skin burn, and the DEGs in each group were screened, including upregulated and downregulated genes. We found that the DEGs were the most in the 4–7 days after burn, which may be because the wound was at the critical point of inflammatory response and tissue regeneration, and the metabolic mechanism in patients was complex. This is similar to the analysis results of Shan *et al*. Using the same dataset, they found that 782 DEGs were identified in the medium term (4–7 days after burn), which is the highest among the three periods.^18^ In addition, using the pig skin burn model, it was found that the inflammatory sites and skin injury sites began to decrease on day 4.^19^ Then, we took the intersection of the differential genes screened by the three groups and found that 432 genes were upregulated in the three groups of skin burn tissues and 351 genes were downregulated in the three groups of skin burn tissues, indicating that these genes may be an important factor for tissue healing after skin burn. This is different from the number of genes screened by Li *et al*. through the construction of burn animal model, which may be caused by species and different exogenous factors.^20^ In short, it is meaningful for us to analyze the DEGs after skin burn in three periods and screen the common genes of the three groups of DEGs as candidate genes.

In order to understand the function and metabolic pathway of the selected candidate genes, GO function analysis method and KEGG pathway enrichment analysis were used. The results showed that the upregulated genes were related to immunity and the downregulated genes were related to skin development and repair after skin burn. The change of immune response has always been paid attention to after skin burn. Skin burn will first destroy the immune system, but the body will self-regulate in a short time to resist environmental microbial infection, improve immunity, and enhance the expression of inflammatory factors and related genes.^21^ NF-KB-dependent signaling pathways, TNF signaling pathways, and IL-17 signaling pathways have long been found to play an important role in skin burns. This study also shows the same results.^22,23^ In addition, skin burns can destroy the development and repair function of skin tissue, which is also shown in our results. At present, many studies are focusing on how to restore and improve the regenerative ability of skin. It has been found that by regulating bFGF, VEGF, TGF - ß 1, and α-SMA gene expression can improve angiogenesis and tissue healing.^24^ However, there are few studies on the relevant molecular mechanisms. This study has enriched the relevant functional pathways, which may provide a theoretical basis for restoring the ability of skin regeneration.

Then we screened the pivotal genes of skin burn-related immune response and skin regeneration through Lasso, and obtained four pivotal genes related to immunity, namely S100A8, UPP1, MMP1, and MMP3, and seven pivotal genes of skin regeneration, namely NR3C2, HSPB3, DPT, NNC1, TMEM255A, DKK2, and SERTM1. There was no difference in the expression of S100A8, but UPP1, MMP1, and MMP3 may become the target genes of skin burn. MMP1 gene has been focused on skin treatment, and it has been proved that the expression of MMP1 is associated with skin tissue morphology and the quality of cultured skin substitutes.^25^ MMP3 is related to the interaction between Guardian cells and keratin in skin regeneration and promotes skin vascular complex allograft rejection.^26,27^ UPP1 has not been reported in skin burn-related fields. Therefore, the research on pivotal immune genes related to skin burn needs to be further explored and verified. In addition, the pivotal genes of skin regeneration screened by us only have DPT expression differences in the verification process, but the DPT expression differences and related studies have not been reported. NR3C2, HSPB3, TNNC1, TMEM255A, DKK2, and SERTM1 showed no expression difference in the secondary validation, which may be related to the difference of accepted samples, the degree of burn and different stages, and need to be further studied and verified. In addition, we also predicted the regulatory network of pivotal genes (TF and lncRNA-miRNA-mRNA) and constructed the regulatory network, which will provide a reference for the research of pivotal genes in the future.^20^

Immune system is one of the most concerned targets in the study of physiological changes after skin burn and the treatment of skin burn. Studies have found that severe burns can disrupt the immune system. Mononuclear phage system (MPS), an important component of the immune system, is the first cellular reactor to respond after burns, and it is also the pivotal to wound sterilization, detoxification, and initiation of adaptive immune response.^28^ However, there is no systematic report on the changes of immune cells after skin burn. This study explored the changes of 24 immune-related gene sets in skin burn tissues. The results showed that there were significant differences in nine immune cells such as Neutrophils, Mast cells, DC, Tcm, Cytotoxic cells, Tgd, aDC, Th1 cells, and NK CD56dim cells between skin burn tissues and normal tissues. Among them, neutrophils were considered in previous reports to prevent bacterial infection in the early stage after skin burn.^29^ Mast cells have a positive effect on the healing process in the mouse skin burn model, and may play a role in skin fibrous diseases.^30^ There are few studies on other immune-related cells in skin burn. In order to further understand the relationship between the skin burns related pivotal genes we screened and these immune cells, we made correlation comparison. The results showed that all differential immune cells, except Tgd, had significant correlation with skin burn-related pivotal genes. These results suggest that the pivotal genes we screened may participate in a certain link of the immune system in skin burn or be regulated by immune response, which may become the direction of future research.

In conclusion, our study has proved that there are differences in the expression of immune-related genes UPP1, MMP1, MMP3, and skin regeneration-related gene DPT in skin burn patients, and there are rich TF regulatory networks and lncRNA-miRNA-mRNA regulatory networks. The differential expression of these genes is closely related to immune cells Neutrophils, Mast cells, DC, Tcm, Cytotoxic cells, aDC, Th1 cells, and NK CD56dim cells, which may provide direction for the research of skin burn and provide targets and clinical indicators for skin burn treatment. However, this study only used different expression profiles of mRNA in burned tissues to obtain key genes related to burns through preliminary screening and verification, without going into proteins. These results also need to be validated in more cell experiments in order to better apply to clinical treatment and drug development. The expression of UPP1, MMP1, MMP3, and DPT in clinical patients with skin burn, the mechanism of action in the treatment of skin burns and the in vivo experiments to confirm their activity remain to be further studied.

## Notes

*Author Contributions*: Hengbo Zhu and Gaofei Zhang participated in the design of this study, and both performed the statistical analysis. Wuquan Li conducted the study, together with Wende Cao collected important background information, Jinglin Zhang and Hong Wang revised the manuscript. All authors approved the final version of the manuscript.

*Funding*: This work was supported by the National Natural Science Foundation of China Regional Science Foundation Grant (Number 81660321).
